# Prevalence and correlates of criminal victimization in Taiwanese outpatients with severe mental illness: a cross-sectional study

**DOI:** 10.3389/fpsyt.2026.1921017

**Published:** 2026-08-27

**Authors:** Chien-Chi Hsu, Chun-Kai Fang, Ying Lin, Ching-Ho Hu, Shu-I Wu

**Affiliations:** 1 Department of Psychiatry, MacKay Memorial Hospital, Taipei, Taiwan; 2 Department of Medicine, MacKay Medical University, New Taipei, Taiwan; 3 Taiwan Alcohol Abstinence and Addiction Prevention Center, Taipei, Taiwan; 4 Institute of Long-Term Care, MacKay Medical University, New Taipei, Taiwan; 5 Department of Thanatology and Health Counseling, National Taipei University of Nursing and Health Sciences, Taipei, Taiwan; 6 Department of Death Care Service, MacKay Junior College of Medicine, Nursing, and Management, Taipei, Taiwan

**Keywords:** bipolar disorder, crime victimization, depression, schizophrenia, severe mental illness

## Abstract

**Background:**

Individuals with severe mental illness (SMI), including schizophrenia and bipolar disorder, are at increased risk of criminal victimization. However, evidence from Asian populations remains limited. This study examined the prevalence of criminal victimization and its associated sociodemographic and clinical factors among Taiwanese adults with SMI.

**Methods:**

In this cross-sectional study, 798 adults with schizophrenia (39.8%) or bipolar disorder (60.2%) who had received outpatient psychiatric care for at least one year at a medical center in northern Taiwan were recruited. Data on past-year criminal victimization, sociodemographic characteristics, and clinical variables were collected. Depressive symptoms, demoralization, medication adherence, and personal and social functioning were assessed using the Patient Health Questionnaire-9 (PHQ-9), Demoralization Scale, Medication Adherence Rating Scale, and Personal and Social Performance Scale, respectively. Univariate analyses and multivariable logistic regression were performed to identify factors associated with criminal victimization.

**Results:**

Overall, 25.1% of participants (n = 200) reported experiencing criminal victimization during the previous year. Living with family members (odds ratio [OR] = 0.56, 95% confidence interval [CI] = 0.37–0.85, p = 0.004) and a diagnosis of bipolar disorder rather than schizophrenia (OR = 0.66, 95% CI = 0.45–0.98, p = 0.035) were associated with lower odds of victimization. Higher odds were associated with a history of suicide attempts (OR = 1.93, 95% CI = 1.35–2.75, p = 0.001), smoking (OR = 1.69, 95% CI = 1.18–2.43, p = 0.004), criminal history (OR = 1.70, 95% CI = 1.06–2.73, p = 0.029), prior psychiatric hospitalization (OR = 1.58, 95% CI = 1.15–2.18, p = 0.015), and greater depressive symptom severity (OR per one-point increase on the PHQ-9 = 1.05, 95% CI = 1.03–1.08, p < 0.001).

**Conclusion:**

Criminal victimization was common among Taiwanese adults with SMI, affecting one in four participants. Victimization was associated with several potentially modifiable clinical factors, particularly depressive symptoms, prior suicide attempts, and psychiatric hospitalization, while family cohabitation appeared to be protective. These findings highlight the importance of incorporating victimization risk assessment into routine psychiatric care and developing preventive interventions that strengthen family support and address modifiable clinical vulnerabilities.

## 1 Introduction

Individuals with severe mental illness (SMI) experience disproportionately high rates of criminal victimization, yet this burden remains underrecognized in both clinical practice and public discourse (1, 2). Although no single universally accepted definition of SMI exists, schizophrenia-spectrum disorders and bipolar disorder are the diagnostic groups most consistently included in epidemiological and community psychiatry research because they are characterized by persistent or recurrent illness, substantial functional impairment, and long-term treatment needs (3, 4). Accordingly, the present study operationally defines SMI as schizophrenia-spectrum disorders and bipolar disorder (3, 5). Despite longstanding public concern regarding violence perpetrated by people with SMI (6), accumulating evidence indicates that they are considerably more likely to become victims than perpetrators of crime (1, 2, 7). As psychiatric care has progressively shifted from long-term institutionalization toward community-based treatment, protecting individuals with SMI from community victimization has become an increasingly important public health and mental health priority (8). Criminal victimization has been associated with poorer quality of life, psychiatric relapse, impaired social functioning, and delayed recovery, thereby compounding the long-term burden of severe mental illness (9–11).

Despite growing recognition of this problem, the true burden of criminal victimization among individuals with SMI remains difficult to estimate. Official police and judicial statistics capture only a fraction of criminal incidents because many victims never report their experiences—a phenomenon widely referred to as the “dark figure of crime” (7, 8, 12). Underreporting appears particularly common among individuals with SMI, who often encounter barriers to disclosure, including anticipated stigma, concerns about not being perceived as credible witnesses, and reluctance to engage with law enforcement (13, 14). Consequently, studies relying solely on official crime records are likely to underestimate both the prevalence of criminal victimization and the vulnerability of this population (8).

Several interrelated clinical and sociocultural mechanisms may contribute to both the elevated risk and the under recognition of criminal victimization among individuals with SMI. Clinically, depressive symptoms are highly prevalent in schizophrenia and bipolar disorder and have consistently been associated with an increased risk of victimization. Depression may be associated with impaired appraisal of environmental and interpersonal cues, reduce perceived self-efficacy, and promote social withdrawal. In addition, depressive cognition has long been linked to learned helplessness, which may be linked to reduced active coping and limited effective responses to threatening situations, thereby associated with heightened vulnerability to victimization (9, 10, 15–17). Moreover, depressive symptoms may be related to diminished motivation to seek help or report victimization after it occurs, further contributing to the “dark figure of crime” (8, 17, 18). Together, these processes may create a self-perpetuating cycle in which depression is associated with increased vulnerability to victimization, while victimization is linked to aggravated psychological distress and functional impairment (1, 9, 18–20).

Sociocultural context may also shape both exposure to victimization and the likelihood of reporting it. In collectivist societies, maintaining family reputation and social harmony often takes precedence over individual disclosure. Individuals with SMI may therefore hesitate to report victimization because they fear stigma or bringing shame upon their families. Consequently, families may try to hide the existence of a member with SMI and hesitate to seek professional help or access appropriate services due to the fear of social stigma and bringing shame upon their families (21, 22). This phenomenon may be particularly salient in East Asian societies, where the concept of face-saving remains deeply embedded in interpersonal relationships (23). At the same time, collectivist cultures may confer important protective effects. In Taiwan, family members commonly assume long-term caregiving responsibilities by providing daily supervision, emotional support, and practical assistance, thereby functioning as “capable guardians” that may reduce exposure to community victimization (24). Understanding this dual influence of collectivist culture—simultaneously discouraging disclosure while strengthening family-based protection—is therefore essential when interpreting criminal victimization among Taiwanese individuals with SMI.

Reported prevalence rates of criminal victimization among individuals with SMI vary widely across international studies, ranging from 4.3% over a one-month period to as high as 56% annually (1, 2, 25, 26). This wide variation is largely attributable to methodological heterogeneity, including differences in study settings, recall periods, victimization assessment methods, and societal crime rates and social welfare systems (2, 7, 24–27). Nevertheless, an important geographical knowledge gap remains. Most existing evidence has been generated in North America and Europe, whereas research from Asian populations remains comparatively limited. Available Asian studies have generally involved relatively small samples, mixed inpatient and outpatient populations, or non-standardized victimization measures, limiting the identification of clinically meaningful risk and protective factors among community-dwelling outpatients with SMI (24, 28). Consequently, large-scale studies using standardized assessments are needed to better characterize the prevalence and correlates of criminal victimization within Asian community psychiatric settings.

To address these knowledge gaps, the present study investigated the one-year prevalence of criminal victimization and examined its sociodemographic and clinical correlates among a large sample of Taiwanese outpatients with schizophrenia-spectrum disorders and bipolar disorder. Guided by Lifestyle–Routine Activity Theory (L-RAT) and previous empirical evidence (29), we examined both clinical vulnerability and sociocultural protection as potential correlates of victimization. We hypothesized that (1) greater clinical severity, reflected by depressive symptom severity, previous suicide attempts, and psychiatric hospitalization history, would be associated with higher odds of past-year criminal victimization; and (2) living with family members would be associated with lower odds of victimization, reflecting the protective role of family guardianship within the Taiwanese sociocultural context.

## 2 Methods

### 2.1 Study design and participants

This cross-sectional survey was conducted between March and December 2013 using a convenience sampling design. Participants were recruited from the psychiatric outpatient clinic of MacKay Memorial Hospital, a major tertiary medical center in Taipei, Taiwan, which provides approximately 8,000 psychiatric outpatient visits each month.

The use of convenience sampling may have introduced selection bias. Because participants were recruited during routine outpatient visits, individuals experiencing acute psychotic exacerbations, those who had disengaged from psychiatric care because of poor treatment adherence, homeless individuals, and those who were institutionalized were unlikely to be represented. Consequently, the study sample reflects a broad spectrum of community-dwelling psychiatric outpatients receiving care at a tertiary medical center serving both urban and suburban populations in northern Taiwan, rather than the full spectrum of individuals with SMI. Therefore, caution is warranted when generalizing these findings to populations with different patterns of healthcare utilization, clinical severity, or service settings.

### 2.2 Inclusion and exclusion criteria

Because no universally accepted diagnostic definition of SMI exists, this study adopted a commonly used operational definition comprising schizophrenia-spectrum disorders and bipolar disorder, consistent with epidemiological studies and community psychiatry research (1, 3–5, 26). These disorders are the diagnostic groups most consistently included in SMI research because they are characterized by recurrent or persistent illness, substantial functional impairment, and long-term treatment needs.

Participants were required to meet the following criteria:

1. Medical record confirmation of schizophrenia, related psychoses (ICD-9-CM (30): 295, 297), or bipolar disorder (ICD-9-CM: 296.0-296.1, 296.4-296.9). At the time of data collection, the ICD-9-CM was the mandatory diagnostic coding system within Taiwan’s National Health Insurance (NHI) registry. Notably, the core conceptualizations of these primary diagnoses remain robustly consistent across the ICD-9-CM, DSM-5, and ICD-11 frameworks.
2. Diagnosis and treatment regimen stable for >1-year preceding enrollment.
3. Minimum age of 20 years at enrollment.
4. Proficiency in spoken Mandarin or Taiwanese and literacy in written Chinese.
5. Provided written informed consent prior to participation.

Excluded if participants met any of the following criteria at the time of enrollment:

1. Presence of acute psychotic symptoms.
2. Significant cognitive impairment.
3. Evident risk of suicide or violence.

### 2.3 Participant recruitment and ethical approval

Potential participants were identified by reviewing outpatient medical records based on the inclusion criteria. A research nurse approached eligible individuals, explained the study’s purpose, and obtained written informed consent prior to participation. A total of 798 individuals consented and participated. The Institutional Review Board (IRB) of MacKay Memorial Hospital approved this study (Approval No. 12MMHIS141).

### 2.4 Research tools

The study utilized the following instruments: a sociodemographic and clinical information, the Taiwan Crime Victimization Survey (TCVS) (24, 31), the Medication Adherence Rating Scale (MARS) (32, 33), the Demoralization Scale (DS) (34–36), the Patient Health Questionnaire (PHQ-9) (37, 38), and the Personal and Social Performance scale (PSP) (39–41).

1. Sociodemographic and clinical data: This form collected demographic and clinical data. Demographic information included gender, age, educational level, occupation, marital status, living situation, and possession of a low-income certificate, disability certificate, or major psychiatric illness card. Clinical information included criminal record history, duration of illness, number of hospitalizations, diagnosis, alcohol use patterns, smoking status, and use of long-acting injectable antipsychotics. Demographic data was self-reported by participants, while clinical data was extracted from medical records by researchers. Harmful drinking was defined according to previous research as consuming alcohol more than three times per week (42).
2. TCVS was developed by Taiwan’s Ministry of Justice and National Police Agency (31) and based on the U.S. National Crime Victimization Survey (NCVS) (12), the TCVS assesses victimization experiences. Using interviews and questionnaires, it probes personal or household victimization, including violent and property crimes, within the past year. Similar methods have previously assessed personal victimization among individuals with SMI in Taiwan (24). This study focused on personal experiences of property and violent crimes over the past year, specifically asking about incidents of theft, assault, robbery, mugging, kidnapping, intimidation, rape, sexual assault, fraud, and identity theft, including the frequency of these occurrences.
3. The MARS: A validated 10-item self-report scale measuring medication-taking behavior over the past week (score range: 0–10; higher scores indicate better adherence). Chinese-version reliability and validity have been confirmed (33).
4. The DS: A 24-item instrument quantifying existential distress (score range: 0–96; scores ≥ 30 indicate moderate-to-severe demoralization). Psychometric properties of the Chinese version are well established (36).
5. The PHQ-9: A 9-item screening tool for depressive symptom severity over the preceding two weeks (score range: 0–27; scores ≥ 10 indicate moderate-to-severe depression). The validated Chinese version was used (38).
6. The PSP: A clinician-rated instrument assessing social functioning across four domains—socially useful activities, personal and social relationships, self-care, and disturbing or aggressive behaviors (score range: 1–100). The Chinese version demonstrates high reliability and validity (41).

### 2.5 Statistical analysis

All statistical analyses were conducted using IBM SPSS Statistics for Windows, Version 25.0 (IBM Corp., Armonk, NY (43)). Descriptive statistics were used to summarize participants’ sociodemographic and clinical characteristics. The normality of continuous variables was assessed using the Kolmogorov–Smirnov test. Because continuous variables were not normally distributed, differences between participants with and without past-year criminal victimization were examined using the Mann–Whitney U test. Categorical variables were compared using Pearson’s chi-square test or Fisher’s exact test, as appropriate.

Univariate analyses were conducted as an exploratory screening procedure to identify candidate variables for multivariable analysis rather than as formal hypothesis tests. Variables demonstrating statistical significance in univariate analyses (p < 0.05), together with variables considered clinically important based on previous literature and *a priori* hypotheses, were entered simultaneously (Enter method) into a multivariable binary logistic regression model to identify independent correlates of past-year criminal victimization while controlling for potential confounding among predictors.

Prior to model interpretation, the assumptions of logistic regression were evaluated. Multicollinearity was assessed using variance inflation factors (VIFs), with all values below 2.5 (range, 1.08–1.85; mean, 1.32), indicating no evidence of problematic multicollinearity. The assumption of linearity in the logit for continuous predictors was examined using the Box–Tidwell procedure. The interaction between PHQ-9 scores and their natural logarithm was not statistically significant (p = 0.412), supporting the linearity assumption. Influential observations were assessed using Cook’s distance, and no cases demonstrated undue influence (all Cook’s distances < 0.05).

Model performance was evaluated using the Hosmer–Lemeshow goodness-of-fit test, Nagelkerke’s pseudo-R², overall classification accuracy, and the area under the receiver operating characteristic curve (AUC). All statistical tests were two-tailed, and statistical significance was defined as p < 0.05.

## 3 Results

### 3.1 Sample characteristics

Of the 798 participants, 34.6% were male (n = 276) and 65.4% female (n = 522), with a mean age of 43.35 years (SD = 11.27; range 20–83), with majority unmarried (73.7%, n = 588) and lived with family (77.4%, n = 618).

### 3.2 Clinical characteristics

Diagnoses included schizophrenia in 39.8% (n = 318) and bipolar disorder in 60.2% (n = 480), with a mean illness duration of 9.98 years (SD = 5.58; range 3–49). Government-issued certifications comprised a major psychiatric illness card from the National Health Insurance Administration (61.5%) and a mental disability certificate from the Social Affairs Bureau (52.3%); 24.9% had at least one comorbid chronic physical illness. Histories of criminal conviction, violent behavior, and suicide attempts were reported by 12.3%, 29.1%, and 48.5%, respectively.

### 3.3 Treatment history

More than half of participants (56.1%, n = 448) had experienced at least one acute psychiatric ward admission; 23.1% (n = 184) had been admitted to a day hospital, and 45.4% (n = 362) had received long-acting injectable antipsychotics.

### 3.4 Baseline assessment scores

Mean baseline scores were: PHQ-9, 10.92 (SD = 7.70); Demoralization Scale, 47.02 (SD = 19.69); PSP, 68.16 (SD = 12.37); and MARS, 6.06 (SD = 2.36).

### 3.5 Lifestyle factors

Current smoking was reported by 38.1% of participants (n = 304), and 3.9% (n = 31) met criteria for harmful alcohol use (42).

### 3.6 Victimization experiences

In the past year, 25.1% (n = 200) of participants reported victimization: 18.0% (n = 144) experienced property crime and 15.9% (n = 127) violent crime. Notably, 71 participants (8.9% of the total sample; 35.5% of those victimized) endured both types of crime. Additional details are provided in **Table 1**.

**Table 1:** Sociodemographic and clinical characteristics (N = 798).

Characteristics	N, (%)
Gender	
Male	276, (34.6%)
Female	522, (65.4%)
Marriage	
Unmarried	588, (73.7%)
Married	210, (26.3%)
Work	
Unemployment	331, (41.5%)
Employment	346, (43.4%)
Household	60, (7.5%)
Student	22, (2.8%)
Retirement	39, (4.9%)
Low-income household	
No	614, (76.9%)
Yes	184, (23.1%)
Living with family	
Without	180, (22.6%)
With	618, (77.4%)
Disability certificate	
No	381, (47.7%)
Yes	417, (52.3%)
Certificate of major psychiatric illness issued under taiwan’s national health insurance	
No	307, (38.5%)
Yes	491, (61.5%)
Psychiatric hospitalization history	
Without	350, (43.9%)
With	448, (56.1%)
Day hospital history	
Without	614, (76.9%)
With	184, (23.1%)
History of suicide	
No	411, (51.5%)
Yes	387, (48.5%)
History of violence	
No	566, (70.9%)
Yes	232, (29.1%)
History of long-acting injection	
No	436, (56.4%)
Yes	362, (45.4%)
Physical illness	
No	599, (75.1%)
Yes	199, (24.9%)
Smoking	
No	494, (61.9%)
Yes	304, (38.1%)
Harmful drinking (Drinking > 3 times/week)	
No	767, (96.1%)
Yes	31, (3.9%)
History of legal problems	
No	700, (87.7%)
Yes	98, (12.3%)
Diagnosis	
Schizophrenia	318, (39.8%)
Bipolar disorder	480, (60.2%)
Education	
Elementary School	67, (8.4%)
Middle School	107, (13.4%)
High School	297, (37.2%)
Undergraduate	294, (36.8%)
Graduate School	33, (4.1%)
Age	43.35 ± 11.27 years old
Duration of severe mental illness	9.98 ± 5.58 years
PHQ9^a^	10.92 ± 7.70
DS^b^	47.02 ± 19.67
MARS^c^	6.06 ± 2.36
PSP^d^	68.16 ± 12.37
Victimization	
No	598, (74.9%)
Yes	200, (25.1%)
Non-violent victimization	
No	654, (82.0%)
Yes	144, (18.0%)
Violent victimization	
No	671, (84.1%)
Yes	127, (15.9%)

^a^Patient Health Questionnaire, ^b^Demoralization Scale, ^c^Medication Adherence Rating Scale, ^d^Personal and Social Performance scale.

### 3.7 Univariate analysis of factors associated with victimization

Univariate analyses showed that, compared with non-victimized participants, those who experienced victimization in the past year were significantly more likely to be unmarried or not cohabiting (27.7% vs. 17.6%, p = .004), have low-income status (34.2% vs. 22.3%, p = .001), not live with family (37.8% vs. 21.4%, p <.001) and hold a mental disability certification (30.5% vs. 19.2%, p <.001). They also had higher rates of psychiatric ward admission (29.9% vs. 18.9%, p <.001), suicide attempts (34.9% vs. 15.8%, p <.001), violent behavior (35.8% vs. 20.7%, p <.001), smoking (35.5% vs. 18.6%, p <.001), harmful alcohol consumption (45.2% vs. 24.3%, p = .008) and criminal convictions (45.9% vs. 22.1%, p <.001).

### 3.8 Comparison of scale scores between victimized and non-victimized groups

Victimized participants exhibited significantly more severe symptoms and poorer functioning than their non-victimized counterparts. Specifically, Mann-Whitney U tests revealed that the victimized group scored significantly higher on the PHQ-9 (Z =-6.098, p <.001) and on the Demoralization Scale (Z = -5.623, p <.001), indicating greater de-pression and demoralization. They also demonstrated worse treatment adherence, with lower MARS scores (Z =-3.815, p <.001), and diminished social functioning on the PSP (Z =-3.909, p <.001). Other details are provided in **Table 2**.

**Table 2:** Comparison of sociodemographic and clinical characteristics by victimization status (N = 798).

Characteristics	Not victimized 74.9% (N:598)	Victimized 25.1% (N:200)	Statistics	P
Gender			χ^2^ = 0.514	.474
Male	76.4%, 211	23.6%, 65		
Female	74.1%, 387	25.9%, 135		
Marriage			χ^2^ = 8.408	.004^*^
Unmarried	72.3%,425	27.7%,163		
Married	82.4%,173	17.6%,37		
Work			χ^2^ = 5.531	.237
Unemployment	71.6%,237	28.4%,94		
Employment	75.7%,262	24.3%,84		
Household	81.7%,49	18.3%,11		
Student	77.3%,17	22.7%,5		
Retirement	84.6%,33	15.4%,6		
Low-income household			χ^2^ = 10.722	.001^*^
No	77.7%,477	22.3%,137		
Yes	65.8%,121	34.2%,63		
Living with family			χ^2^ = 20.008	.000^*^
Without	62.2%,112	37.8%,68		
With	78.6%,486	21.4%,132		
Disability certificate			χ^2^ = 13.525	.000^*^
No	80.8%,308	19.2%,73		
Yes	69.5%,290	30.5%,127		
Major psychiatric Illness certification			χ^2^ = 0.689	.407
No	76.5%,235	23.5%,72		
Yes	73.9%,363	26.1%,128		
Psychiatric hospitalization history			χ^2^ = 12.783	.000^*^
Without	81.14%,284	18.86%,66		
With	70.09%,314	29.91%,134		
Day hospital history			χ^2^ = 1.783	.182
Without	76.1%,467	23.9%,147		
With	71.2%,131	28.8%,53		
History of suicide			χ^2^ = 38.589	.000^*^
No	84.2%,346	15.8%,65		
Yes	65.1%,252	34.9%,135		
History of violence			χ^2^ = 20.217	.000
No	79.3%,449	20.7%,117		
Yes	64.2%,149	35.8%,83		
History of long-acting injection			χ^2^ = .288	.591
No	75.7%,330	24.3%,106		
Yes	74.0%,268	26.0%,94		
Physical illness			χ^2^ = .936	.333
No	75.8%,454	24.2%,145		
Yes	72.4%,144	27.6%,55		
Smoking			χ^2^ = 28.628	.000^*^
No	81.4%,402	18.6%,92		
Yes	64.5%,196	35.5%,108		
Harmful drinking			χ^2^ = 6.937	.008^*^
No	75.7%,581	24.3%,186		
Yes	54.8%,17	45.2%,14		
History of legal problems			χ^2^ = 25.874	.000^*^
No	77.9%,545	22.1%,155		
Yes	54.1%,53	45.9%,45		
Diagnosis			χ^2^ = .003	.960
Schizophrenia	74.8%,238	25.2%,80		
Bipolar disorder	75.0%,360	25%,120		
Education			χ^2^ = 1.778	.776
Elementary School	70.1%,47	29.9%,20		
Middle School	75.7%,81	24.3%,26		
High School	74.4%,221	25.6%,76		
Undergraduate	75.5%,222	24.5%,72		
Graduate School	81.8%,27	18.2%,6		
Age	43 (16, 29-43)	43 (15.75, 28-43)	Z=-0.412.	.680
Duration of severe mental illness	9 (7, 3-9)	10 (7, 3-10)	Z=-0.317	.751
PHQ9**^a^**	9 (12, 0.9-9)	13.5 (13, 4-13.5)	Z=-6.098	.000^*^
DS**^b^**	44 (28, 20-44)	54 (25.5, 29-54)	Z=-5.623	.000^*^
MARS**^c^**	7 (3, 3-7)	6 (3, 2-6)	Z=-3.815	.000^*^
PSP**^d^**	70 (20, 55-70)	65 (15, 50.1-65)	Z=-3.909	.000^*^

(1) Statistical analysis: Categorical variables were compared using Pearson’s chi-square test (χ²). Continuous variables were evaluated using the Mann-Whitney U test (Z-value) and are presented as medians (interquartile range, Q1–Q3) due to their non-normal distribution. *p < 0.05. (2) ^a^Patient Health Questionnaire; ^b^Demoralization Scale; ^c^Medication Adherence Rating Scale; ^d^Personal and Social Performance scale.

### 3.9 Independent correlates of criminal victimization

Multivariable binary logistic regression identified seven independent correlates of past-year criminal victimization (**Table 3**). Living with family (OR = 0.56, 95% CI = 0.37–0.85, p = .004) and a diagnosis of bipolar disorder, compared with schizophrenia-spectrum disorders (OR = 0.66, 95% CI = 0.45–0.98, p = .035), were independently associated with lower odds of victimization. In contrast, higher odds of victimization were observed among participants with a history of suicide attempts (OR = 1.93, 95% CI = 1.35–2.75, p = .001), current smoking (OR = 1.69, 95% CI = 1.18–2.43, p = .004), prior criminal convictions (OR = 1.70, 95% CI = 1.06–2.73, p = .029), previous psychiatric hospitalization (OR = 1.58, 95% CI = 1.15–2.18, p = .015), and greater depressive symptom severity, with each one-point increase in PHQ-9 score associated with a 5% increase in the odds of victimization (OR = 1.05, 95% CI = 1.03–1.08, p <.001).

**Table 3:** Logistic regression analysis predicting victimization.

Variable	B	SE	p	OR	95%CI
Living with family	-.572	.199	.004^*^	.564	.382~.833
History of Suicide	.659	.192	.001^*^	1.932	1.326~2.815
Smoking	.527	.184	.004^*^	1.694	1.181~2.429
History of legal problems	.533	.244	.029^*^	1.704	1.057~2.748
Bipolar vs. Schizophrenia	-.415	.196	.035^*^	.660	.449~.970
PHQ9^a^ (per unit)	.047	.012	.000^*^	1.049	1.023~1.074
Psychiatric Hospitalization History	.455	.188	.015^*^	1.577	1.092~2.277

^a^Patient Health Questionnaire, *<0.05.

Model Performance & Fit Metrics: Hosmer-Lemeshow *χ*^2^(8) = 6.79, *p* = .560; Nagelkerke *R*^2^ = 0.178; Overall Classification Accuracy = 77.2%; Area Under ROC Curve (AUC) = 0.731 (95% CI = 0.690–0.772, *p* <.001). Multicollinearity checked (all VIF < 2.5). Linearity of logit verified for PHQ-9 (Box-Tidwell test *p* = .412). Influential observations checked (all Cook’s distances < 0.05).

The multivariable binary logistic regression model was constructed using the simultaneous entry (Enter) method. To control for mutual confounding, all variables demonstrating statistical significance (*p* <.05) in the univariate analyses were included in the model. Additionally, primary diagnosis (bipolar disorder vs. schizophrenia) was forced into the model *a priori* due to its core clinical relevance to the study design. For clarity and brevity, this table displays only the independent variables that remained statistically significant in the final model. Variables that were adjusted for but did not reach statistical significance included: marital status, low-income household status, disability certificate status, history of violent behavior, harmful alcohol use, Demoralization Scale (DS) score, Medication Adherence Rating Scale (MARS) score, and Personal and Social Performance (PSP) score.

The final model demonstrated satisfactory performance. The Hosmer–Lemeshow goodness-of-fit test was non-significant (χ²(8) = 6.79, p = .560), indicating good calibration. The model explained 17.8% of the variance in past-year victimization (Nagelkerke’s R² = 0.178), achieved an overall classification accuracy of 77.2%, and demonstrated acceptable discriminative ability (AUC = 0.731, 95% CI = 0.690–0.772, p <.001).

## 4 Discussion

This study aimed to investigate crime victimization experiences among community-dwelling individuals with SMI in Taiwan. Our principal finding revealed a significant burden of victimization: 25.1% of participants reported experiencing a crime within the past year, encompassing violent victimization (15.9%) and non-violent (property) victimization (18.0%). While the elevated prevalence of victimization among individuals with SMI is well-established, much of the existing research originates from western countries, highlighting insufficient data from Asian contexts (24, 25, 28). This study utilizes one of the largest samples to date in Asia employing a standardized crime victimization survey instrument. The overall victimization rate aligns with findings from systematic reviews and meta-analyses, confirming that individuals with SMI face a substantially higher likelihood of experiencing victimization compared to the general population (1). Meta-analyses focusing on psychosis indicated median three-year rates of 20% for violent and 19% for non-violent victimization (25), while studies examining one-year prevalence of violent victimization report rates ranging widely from 7.1% to 56% (2). Our results fall within these established ranges, acknowledging that variations across studies are expected due to differences in recruitment criteria, location, sample size, survey timeframe, and methodology (1, 2, 25). Nonetheless, the consistent finding across diverse research is the heightened vulnerability of this population.

Both *a priori* hypotheses received support from the multivariable findings. As predicted, greater clinical severity showed an independent association with victimization: each additional point on the PHQ-9 was associated with an approximately 5% higher odds (OR = 1.05), a history of suicide attempts was associated with nearly double the odds (OR = 1.93), and prior psychiatric hospitalization was associated with 58% higher odds (OR = 1.58)—collectively lending support to the view that greater clinical burden is linked to elevated vulnerability to victimization. Also as predicted, living with family was associated with 44% lower odds of victimization (OR = 0.56), providing support for the hypothesized association between family cohabitation and reduced victimization risk in this Taiwanese outpatient population.

Beyond these pre-specified associations, the model further identified current smoking (OR = 1.69) and a prior criminal record (OR = 1.70) as additional factors independently associated with higher odds of victimization, along with a diagnosis of bipolar disorder relative to schizophrenia (OR = 0.66) as a factor independently associated with lower odds—extending the range of correlates considered beyond those originally anticipated.

### 4.1 Prevalence in comparative context

Interestingly, our overall victimization rate (25.1%) diverges from a previous, smaller-scale Taiwanese study using similar methods, which reported a rate of 16.8% (24). This discrepancy may reflect a result of differences in sample characteristics and recruitment settings. The previous study included 155 participants, notably 61 individuals hospitalized in acute psychiatric wards at the time of the survey and featured a higher proportion of individuals with schizophrenia (59.4%) (24). In contrast, our larger sample (N = 798) consisted entirely of outpatients, with a lower proportion diagnosed with schizophrenia (39.8%). These variations in sample acuity, diagnostic composition, and living situation (community vs. partly inpatient) likely contributed to the differing observed victimization rates.

### 4.2 Factors positively associated with victimization

This study used multivariate analysis to identify significant correlates of victimization. Positive associations were found for a history of suicide attempts, more severe depressive symptoms, current smoking, a prior criminal record, and previous acute psychiatric hospitalization, while living with family and a diagnosis of bipolar disorder (relative to schizophrenia) were inversely associated with victimization.

#### 4.2.1 Suicide attempts and depressive symptoms

Our findings indicated that a history of suicide attempts was associated with nearly double the odds of victimization (OR = 1.93), while each one-point increase in the PHQ-9 depression score was associated with an approximately 5% increase in the odds of victimization (OR = 1.05). The relationship between depressive symptoms, suicidality, and victimization is complex and likely bidirectional. While victimization experiences are associated with the exacerbation of depressive symptoms and related mental health issues (44, 45), the reverse pathway is also plausible. Pre-existing depressive symptoms—specifically manifesting within the context of schizophrenia and bipolar disorder rather than primary Major Depressive Disorder (MDD)—might be linked to impaired judgment, motivation, or the ability to navigate or exit risky situations, thereby associated with heightened vulnerability (17). This aligns with research showing higher victimization rates among those hospitalized for suicide attempts (45) and findings linking depressive symptoms with recent victimization and increased severity among those with a suicide attempt history (17). Furthermore, twin studies suggest gene-environment correlations may be present, indicating that pre-existing mental health vulnerabilities may share underlying pathways with subsequent victimization (46). Inherent vulnerability, impaired judgment, social isolation, prior trauma, and being perceived as easier targets are possible explanations for the heightened susceptibility of SMI patients.

#### 4.2.2 Smoking status

Current smoking was associated with a 1.7-fold increase in the odds of victimization (OR = 1.69). This resonates with large community studies identifying smokers as having elevated risks for both crime perpetration and victimization (47). Smokers are more likely to reside in or perceive their neighborhoods as high-crime areas (48), and community violence levels are linked to smoking intensity and reduced cessation attempts (49). Thus, smoking might serve as a marker for exposure to higher-risk environments or lifestyles. In addition, factors like greater exposure to deviant peers, potentially impaired judgment, or reduced impulse control among some smokers could partially explain this observed vulnerability (50).

#### 4.2.3 Criminal record

A prior criminal record was associated with a 1.7-fold increase in the odds of victimization (OR = 1.70), consistent with established research demonstrating a strong link between offending behavior and subsequent victimization (1, 25). A meta-analysis specific to individuals with mental illness reported a 4.3-fold increase in victimization odds associated with a criminal history (25). Lifestyle and Routine Activity Theory (L-RAT) (29, 51, 52) offers a framework, suggesting victimization occurs at the confluence of a motivated offender, a suitable target, and the absence of capable guardianship. Individuals with SMI and a criminal record may experience increased exposure to risky situations or offenders (lifestyle/environment), share underlying vulnerabilities associated with both offending and susceptibility to victimization (e.g., impulsivity, substance use), and potentially lack of effective guardianship (51, 52).

#### 4.2.4 History of acute psychiatric hospitalization

A history of acute psychiatric hospitalization was associated with a 1.6-fold increase in the odds of victimization (OR = 1.58), corresponding with prior research suggesting that recent or frequent hospitalizations are linked to higher victimization rates (1). For example, a Danish study found that discharged psychiatric patients had a five times higher 10-year homicide mortality rate (53), and within healthcare systems, hospitalization frequency has been correlated with a greater likelihood of physical victimization (54). A history of acute hospitalization often signifies greater illness severity or chronicity (54), potentially rendering individuals more vulnerable or marking them as more suitable targets in the eyes of perpetrators.

### 4.3 Factors inversely associated with victimization

#### 4.3.1 Living with family

Living with family was inversely associated with victimization (OR = 0.56). According to the L-RAT framework (29, 51, 52), this association suggests that family members can serve as capable guardians, which is associated with a decreased likelihood of such events. This contrasts sharply with the well-documented increased vulnerability associated with homelessness or unstable housing among individuals with SMI (1, 2, 25, 51, 55), where meta-analyses indicate a 2.6-fold increase in victimization odds (55). Living with family likely provides a safer environment and effective supervision, thereby potentially linked to limited exposure and opportunities for victimization.

#### 4.3.2 Bipolar disorder diagnosis

Compared to schizophrenia, a diagnosis of bipolar disorder was associated with a lower odds of victimization (OR = 0.66). Plausible explanations include potentially better average functioning, longer periods of stability, or stronger family connections among individuals with bipolar disorder relative to schizophrenia, which may support better self-protection capabilities. The literature examining specific diagnoses and victimization prevalence presents inconsistent findings. While some studies suggest that any psychiatric symptoms are associated with higher odds of victimization, potentially additively (10), or link specific symptoms like psychosis or mania to these experiences without differentiating primary diagnosis (25), others find no difference between schizophrenia and bipolar disorder (55), or even suggest lower (56) or higher victimization rates for schizophrenia (57). Methodological variations, study populations, and varying controls for confounders like offending behavior likely account for these discrepancies (58). Our finding adds to this complex picture, and should be interpreted cautiously, underscoring the need for further nuanced, longitudinal research.

### 4.4 Interpretation of other scale findings

While univariate analyses linked higher demoralization (DS), poorer medication adherence (MARS), and worse social functioning (PSP), disability certification, and low-income status to victimization, these associations did not remain statistically significant in the multivariate model after accounting for other covariates. This likely reflects substantial clinical overlap, potential multicollinearity, and the influence of con-founding factors and interrelationships between variables. For example, poor medication adherence is associated with higher rates of offending and hospitalization (59, 60), both of which demonstrated significant positive associations with victimization in our model. Similarly, functional impairment (captured by PSP) is often more pronounced in schizophrenia than bipolar disorder (61), aligning with our finding that a diagnosis of bipolar disorder was inversely associated with victimization relative to schizophrenia. Demoralization is closely linked to depression and suicidality (62), which were strong independent correlates in our analysis. Therefore, while not emerging as independent factors in the final model, demoralization, adherence, and social functioning likely intersect with the overall vulnerability profile and remain clinically important indicators of potential susceptibility.

### 4.5 Clinical implications

Collectively, the identified correlates have several practical implications for psychiatric care. Rather than viewing criminal victimization as an unpredictable external event, clinicians may consider victimization risk as an integral component of routine risk assessment among individuals with SMI. Patients presenting with severe depressive symptoms, a history of suicide attempts, repeated psychiatric hospitalizations, or prior legal involvement may warrant more proactive inquiry regarding recent victimization experiences, personal safety, and environmental risks. Incorporating trauma-informed victimization screening into standard outpatient assessments may facilitate earlier identification of vulnerable individuals and enable timely referral to psychosocial, legal, or community support services. Moreover, the protective association of family cohabitation highlights the importance of engaging caregivers in safety planning and community rehabilitation, particularly within East Asian settings where family members often function as primary sources of support and informal guardianship.

### 4.6 Limitations and strengths

Several limitations should be considered when interpreting the present findings. First, the cross-sectional design precludes conclusions regarding temporal or causal relationships between the identified correlates and criminal victimization. For example, depressive symptoms are associated with increased vulnerability to victimization, while victimization itself is associated with the exacerbation of depression and suicidal ideation. Similar bidirectional relationships may also exist for smoking, psychiatric hospitalization, and criminal history. Second, victimization and other clinical variables were assessed retrospectively using self-report measures, which may be subject to recall and social desirability biases. Importantly, although we collected data on participants’ prior criminal convictions from medical records, we did not have access to linked official judicial or police records specifically regarding their victimization status. Consequently, we were unable to directly quantify the magnitude of the “dark figure of crime” (the ratio of reported to unrecorded incidents) within this cohort through statistical cross-referencing. Third, although exploratory univariate analyses involved multiple comparisons, these analyses were intended solely to identify candidate variables for the multivariable model, in which potential confounding was subsequently addressed.

Several additional considerations relate to the generalizability of the findings. The study sample comprised community-dwelling psychiatric outpatients recruited from a single tertiary medical center in Taiwan and therefore may not represent individuals with SMI who are homeless, institutionalized, or disengaged from psychiatric services. Furthermore, because the data were collected in 2013, the study primarily captured traditional forms of property and violent crime and did not assess emerging forms of digital victimization, such as cybercrime or online fraud. Although patterns of criminal victimization may have evolved over the past decade, the principal correlates identified in this study—including depression, suicidal history, psychiatric hospitalization, and family cohabitation—likely reflect enduring clinical and social mechanisms that remain highly relevant to contemporary psychiatric practice.

Despite these limitations, this study has several notable strengths. It represents one of the largest investigations of criminal victimization among community-dwelling individuals with SMI in Taiwan, employed a standardized instrument designed to capture both reported and unreported victimization, and examined a broad range of demographic and clinical correlates. The findings also extend existing literature by highlighting the potential protective role of family cohabitation within an East Asian collectivist context, providing culturally relevant evidence that complements previous studies conducted primarily in Western populations.

## 5 Conclusion

Criminal victimization is common among community-dwelling individuals with SMI in Taiwan, with one in four outpatients reporting at least one victimization incident during the preceding year. This study identified several independent correlates of victimization, including depressive symptom severity, previous suicide attempts, smoking, criminal convictions, psychiatric hospitalization, diagnosis, and family cohabitation. Collectively, these findings suggest that vulnerability to criminal victimization is shaped not only by psychiatric illness itself but also by the interaction of clinical vulnerability, behavioral risk, and social protection.

By identifying several clinically relevant and potentially modifiable correlates, this study contributes to a better understanding of victimization risk among individuals with SMI and provides an evidence base for future prevention strategies. Prospective longitudinal and intervention studies are warranted to clarify causal relationships and determine whether addressing these modifiable factors can reduce victimization and improve long-term recovery.

## Data Availability

The datasets presented in this article are not readily available because the datasets are restricted due to institutional and legal regulations. Data sharing is limited to anonymized information and requires prior approval from the ethics committee. Requests to access the datasets should be directed to C-CH, hsuchienchi@gmail.com.
